# Using the behavior change wheel to develop text messages intervention (MedText-PCI) to promote medication adherence in patients after PCI

**DOI:** 10.3389/fdgth.2026.1727102

**Published:** 2026-05-08

**Authors:** Fang Yong, Han Zhihao, Zhou Xi, Wei Lai, Ni Yingyuan, Jiang Zhili, He Jing, Wang Jiangqian, Hua Jiawei, Wenxiao Wu, Qin Su

**Affiliations:** 1 Changsha Medical University, Changsha, China; 2 Zhejiang Chinese Medical University, Hangzhou, China; 3 Zhejiang Yongkang Health School, Jinhua, China; 4 National University of Defense Technology, Changsha, China; 5 Zhejiang Industry Polytechnic College, Shaoxing, China; 6 The Second Affiliated Hospital of Zhejiang Chinese Medical University, Hangzhou, China

**Keywords:** behavior change techniques, behavior change wheel, coronary heart disease, medication adherence, text message intervention

## Abstract

**Objectives:**

This study aimed to use the Behaviour Change Wheel (BCW) framework to develop a theory-informed text message intervention (MedText-PCI) to improve medication adherence in patients after percutaneous coronary intervention (PCI).

**Methods:**

This intervention development study was guided by the BCW framework. A behavioural diagnosis was first undertaken to identify barriers and facilitators to medication adherence, which were then mapped onto the Capability, Opportunity, Motivation–Behaviour (COM-B) model. Intervention functions and behaviour change techniques (BCTs) were selected systematically using the APEASE criteria. Based on the selected BCTs, text messages were developed collaboratively by medical experts and a communication specialist.

**Results:**

The final MedText-PCI intervention comprised 115 theory-informed text messages incorporating 15 distinct BCTs. The messages addressed key domains including treatment benefits, consequences of non-adherence, action planning, and motivational reinforcement. Iterative expert review supported the clinical accuracy, clarity, and accessibility of the intervention content.

**Conclusion:**

Using the BCW framework, this study developed a theory-informed text message intervention to support medication adherence after PCI. The development process was systematic and transparent, providing a reproducible basis for future feasibility testing and outcome evaluation. MedText-PCI has the potential to inform adherence support strategies in post-PCI secondary prevention.

**Clinical Trial Registration:** https://www.chictr.org.cn/bin/project/edit?pid=172238, identifier ChiCTR2200061353.

## Introduction

Coronary Atherosclerotic Heart Disease (CHD), also known as coronary heart disease, is characterized by the narrowing or blockage of coronary arteries due to atherosclerosis, which results in myocardial ischemia, hypoxia, or necrosis (1). Globally, approximately 170 million people are affected by CHD, making it one of the leading causes of death worldwide. Each year, around 720,000 new cases and 335,000 recurrent cases of CHD are diagnosed (2). Percutaneous coronary intervention (PCI) remains the standard treatment for CHD (3). PCI can temporarily alleviate symptoms by reopening blocked arteries, but it does not stop the progression of coronary atherosclerosis (3). Patients remain at risk for restenosis and cardiovascular events, which often lead to repeated hospital readmissions (4). Studies suggest that the incidence of coronary restenosis following PCI can reach 10.6% (5).

Authoritative guidelines recommend that patients with CHD who meet the criteria and have no contraindications should continue evidence-based drug therapy to control symptoms and reduce cardiovascular risk after PCI (1, 6). Long-term adherence to these treatments is essential for improving patient outcomes. However, medication adherence among PCI patients remains a major concern and contributes to poor prognosis. Data from the China PEACE cohort study indicate that nearly 30% of PCI patients have poor medication adherence (7). One year after PCI, adherence rates to aspirin and P2Y12 inhibitors were 50.5% and 49.9%, respectively (8). Failure to adhere to secondary prevention medications increases the risk of cardiovascular events by 40% and mortality by 48%, posing a significant threat to patient health (9, 10). These findings underscore the importance of effective medication management in improving the prognosis of PCI patients. As the prevalence of CHD continues to rise, so does the number of PCI procedures, highlighting the urgent need for cost-effective and scalable interventions to enhance patient adherence to prescribed medications (11, 12).

With the widespread use of mobile phones, text messaging has become a widely accepted way to communicate. The low cost and minimal time commitment associated with text messaging make it a valuable tool for enhancing behavior change interventions, particularly in supporting medication adherence (13, 14). Indeed, Existing research has demonstrated that text messaging via mobile phones can positively influence health behaviors, including diet (15), weight loss (16), physical activity (17), and medication adherence (18). However, To develop effective, scalable text messaging interventions, it is essential for researchers to define a conceptual model specifying the intervention components and their behavioral mechanisms (19). After defining these components, further studies are needed to determine the optimal frequency, content, and intensity of messages required for meaningful behavioral change. Despite this, many behavioral text messaging programs fail to clearly define the theoretical foundations guiding message design, limiting their transparency and replicability (20, 21). This gap stems from multiple challenges, including limited application of theory in published studies, insufficient methodological frameworks for evaluating and selecting appropriate theories, and the overwhelming diversity of available behavior change theories. As a result, many practitioners lack the necessary guidance to effectively operationalize theoretical principles in intervention development (22).

The Behavior Change Wheel (BCW) offers a comprehensive framework for intervention development, integrating the COM-B model (capability, opportunity, motivation) with a taxonomy of intervention functions and behavior change techniques (BCTs) (23). Applying this framework allows researchers to systematically link patient barriers with practical strategies and to ensure that intervention components are theoretically grounded. While the BCW has been applied to lifestyle interventions such as diabetes prevention, its use in the context of cardiovascular pharmacotherapy is still limited (24–27).

This study describes the development of MedText-PCI, a theory-informed text messaging intervention designed to support medication adherence in patients after PCI. By mapping the specific behavioral challenges of this population onto the BCW framework, we aimed to create a reproducible process for generating targeted and patient-centered messages that can be feasibly implemented in clinical practice.

## Methods

### Theoretical framework for intervention development

**Figure 1:**
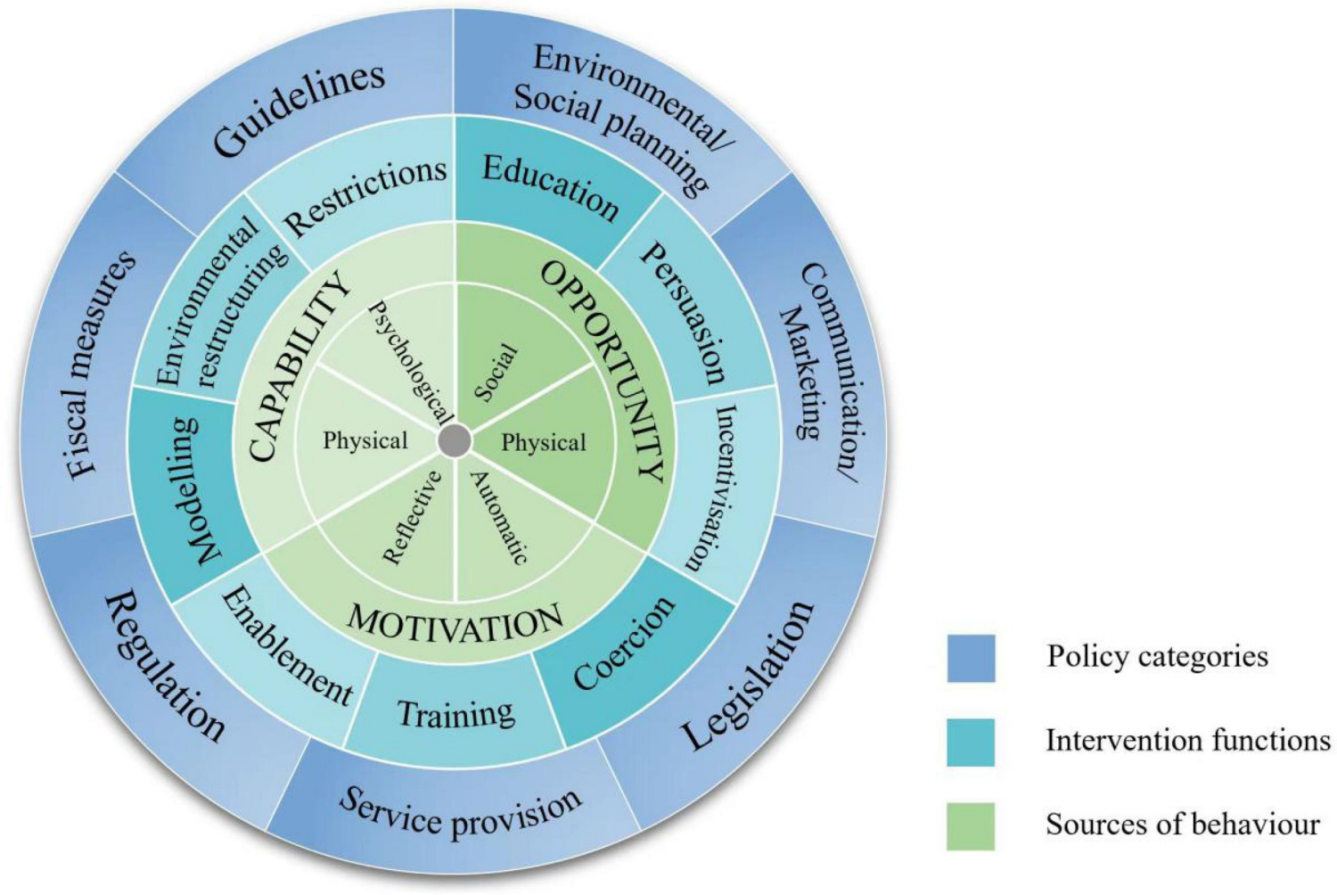
The behavior change wheel.

In developing the intervention, we followed the step-by-step guidance of the BCW (28) (see Figure 1).The BCW synthesises key theoretical constructs from 19 behavioral science frameworks and links them to the COM-B model, which is broad enough to apply to various behaviors in different contexts. It also offers a standardised taxonomy for classifying interventions and understanding the relationships between outcomes and change mechanisms, a significant advance that aids in evaluating, replicating, and comparing interventions.

At the center of the BCW is the COM-B model, which proposes that behavior is shaped through interactions between capability, opportunity, and motivation. By analyzing these elements, researchers can identify whether a target behavior requires improved capability (physical or psychological), enhanced opportunity (social or physical), increased motivation (reflective or automatic), or a combination of these factors.

The second layer of the BCW outlines nine intervention functions, such as education, persuasion, and training, that describe different approaches to influencing behavior. These functions are linked to a comprehensive taxonomy of 93 behavior change techniques (BCTs), which represent the smallest active components that can be implemented within an intervention (29).

Finally, the outermost layer comprises seven policy categories that describe the broader strategies through which intervention functions can be delivered and supported, including guidelines, communication and marketing, and environmental planning. As the behavioral related components of the COM-B model can be addressed through more than one intervention function and policy category, the inner wheels can be ‘moved around’ the centre core of the BCW.

### Application of BCW method to MedText-PCI

**Figure 2:**
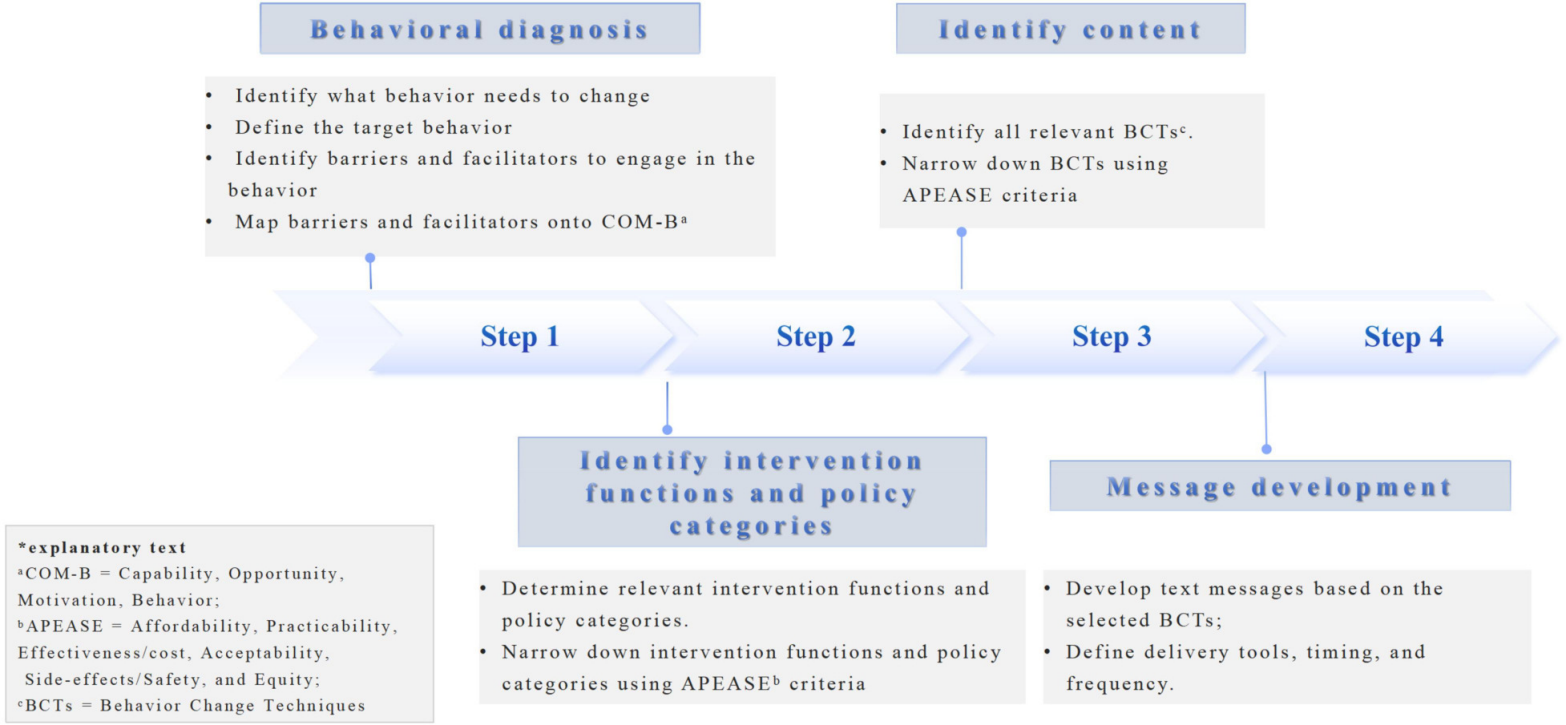
Application of the BCW framework in this study.

The present study applied BCW to systematically guide the development of MedText-PCI targeting medication adherence in post-PCI patients. The BCW is broadly split into three stages (23): (i) understanding the behavior; (ii) identification of intervention options; and (iii) identification of content and implementation options. Because mobile phones are widely used and text messaging is a common, low-cost, and effective tool to promote behavior change, text messaging was chosen as the delivery method at the start of intervention design. In this study, we expanded these into four practical stages to form the initial intervention plan (see Figure 2).

### Step 1. Behavioral diagnosis

Based on guideline recommendations and published evidence, the core behavior of interest was defined as daily intake of prescribed secondary prevention medications without missed or incorrect doses. To contextualize this behavior, we reviewed clinical literature, survey data, and expert consultations. Barriers included forgetfulness, concern about adverse effects, financial stress, and lack of family or physician support. Facilitators included strong risk perception, good health literacy, and regular follow-up. These determinants were categorized into capability, opportunity, and motivation according to the COM-B model. Determinants of adherence were mapped to the three COM-B components. Forgetfulness and health literacy were categorized within capability; financial stress, family or physician support, and regular follow-up within opportunity; and concern about adverse effects and strong risk perception within motivation.

### Step 2. Identify intervention functions and policy categories

Upon completing the behavioural diagnosis, we identified the intervention functions and policy categories most likely to support behaviour change within the COM-B model. We first considered the range of intervention functions proposed in the Behaviour Change Wheel (BCW) framework as candidate options. In the BCW, incentivization refers to creating an expectation of reward, coercion to creating an expectation of cost or punishment, restriction to using rules or constraints to reduce the opportunity to perform a competing behaviour, environmental restructuring to changing the physical or social context, and modelling to providing an example for people to aspire to or imitate. These functions were considered initially as part of the BCW taxonomy rather than assumed to be equally appropriate for a text message intervention in post-PCI medication adherence. Guided by the BCW matrices linking COM-B components to intervention functions, all candidate functions were then appraised using the APEASE (Affordability, Practicability, Effectiveness/cost, Acceptability, Side-effects/Safety, and Equity) criteria to determine which were realistic, acceptable, and suitable for inclusion in the present intervention. In practice, functions judged less feasible or less appropriate for this context, such as coercion or restriction, were not prioritised in the final intervention. The selected intervention functions were subsequently entered into the BCT intervention mapping tables (Supplementary File S1).

We then used the BCW matrix linking intervention functions to policy categories to identify potentially relevant policy categories, including communication/marketing, guidelines, fiscal measures, regulation, legislation, environmental/social planning, and service provision. These were further refined using the APEASE criteria to select those most suitable for the development and implementation of MedText-PCI.

### Step 3. Identify content

After identifying the relevant intervention functions, we determined which BCTs would best support these functions in a text messaging intervention. The matrices within the BCW framework were used to guide the selection and to link BCTs to the chosen intervention functions. BCTs appropriate for each function are detailed in the BCW (23). To systematically identify which BCTs were most likely to influence the target behaviors, we reviewed current literature (25, 30–38).

After compiling the list of potentially relevant BCTs, each technique was reviewed using the APEASE framework to determine its feasibility and relevance. The assessment considered whether the technique could be implemented within reasonable resource limits, whether it was practical for large-scale delivery in the form of short text messages tailored to post-PCI patients, and whether its expected benefits justified the associated costs. In addition, attention was given to the likelihood that the selected techniques would be understandable and engaging for patients, with further acceptability testing planned in subsequent stages. Consideration was also given to the potential impact on health equity, aiming to ensure that the intervention would reduce rather than exacerbate disparities. Through this process, a final set of BCTs was established to guide the development of the text message library for improving medication adherence in post-PCI patients.

### Step 4. Message development

Message development was based on the previously identified BCTs. Drafts were prepared by ZX and WL, trained in BCT identification, and reviewed by FY and HZH, who have expertise in coronary heart disease and behavioral research. Each message was limited to 160 characters, the maximum supported by most mobile phones, to ensure accessibility for patients without smartphones, as smartphone-only interventions may increase health disparities (39). The wording was kept concise and free of jargon or abbreviations to reduce ambiguity. Readability was checked with the Flesch-Kincaid grade level test, and messages above the eighth-grade level were revised in line with recommendations for adult health communication (40). After these adjustments, professional reviewers confirmed clarity and accuracy.

For fidelity assessment, an independent reviewer (WWX), trained in BCT identification but not involved in drafting, coded all messages to verify consistency between intended and delivered techniques. Discrepancies were resolved through consensus discussions with WL, resulting in a finalized library in which each message accurately reflected the targeted BCTs.

## Results

### Step 1. Behavioral diagnosis

Based on guideline recommendations for post-PCI pharmacological management, we conducted a behavioral diagnosis under the guidance of the BCW framework to identify the final target behavior (Table 1). Specifically, the behavior of interest was defined as taking medication daily without missing or incorrect doses. The target group for this behavior was post-PCI patients, who are at high risk for recurrent cardiovascular events if adherence is suboptimal. The behavior was specified to be performed every day after PCI, emphasizing consistency in medication intake as a critical component of secondary prevention. This behavior was expected to occur in various everyday contexts, including at home, at work, and within community settings. Detailed information on this target behavior specification is provided in Table 1. These specifications provided a clear and actionable foundation for the subsequent development of the text message intervention aimed at addressing the key barriers and facilitators related to medication adherence in this patient population. The identification of barriers and facilitators to engaging in the behavior, and their mapping onto the COM-B model, have been described in detail in our previous publication (41).

**Table 1:** Specification of the target behavior.

Target behavior	Medication non-adherence
Actor	Post-PCI patients
Action	Taking medication daily without missing or incorrect doses
Time and frequency	Every day after PCI
Context	Home, workplace, community

### Step 2. Identify intervention functions and policy categories

All nine intervention functions and seven policy categories were initially identified as potentially relevant to address the barriers and facilitators related to medication adherence in post-PCI patients. Each of these candidate intervention options was evaluated using the APEASE criteria. As a result, six intervention functions (education, persuasion, training, incentivization, environmental restructuring, and enablement) were identified as appropriate for inclusion in a text messaging intervention for post-PCI patients. The remaining intervention functions were excluded because they were either inconsistent with the overall communication style suitable for post-PCI patients (restriction and coercion) or could not be effectively incorporated into brief text messages of less than 160 characters. The selected intervention functions were incorporated into the BCT mapping tables (Supplementary File S1). One policy category (communication/marketing) was identified as appropriate for implementation. The remaining policy categories (guidelines, environmental/social planning, legislation, service provision, regulation, and fiscal measures) were excluded because they were not feasible or applicable in this context (i.e., these categories could not be effectively targeted through text messaging delivery to this patient population).

### Step 3. Identify content

**Figure 3:**
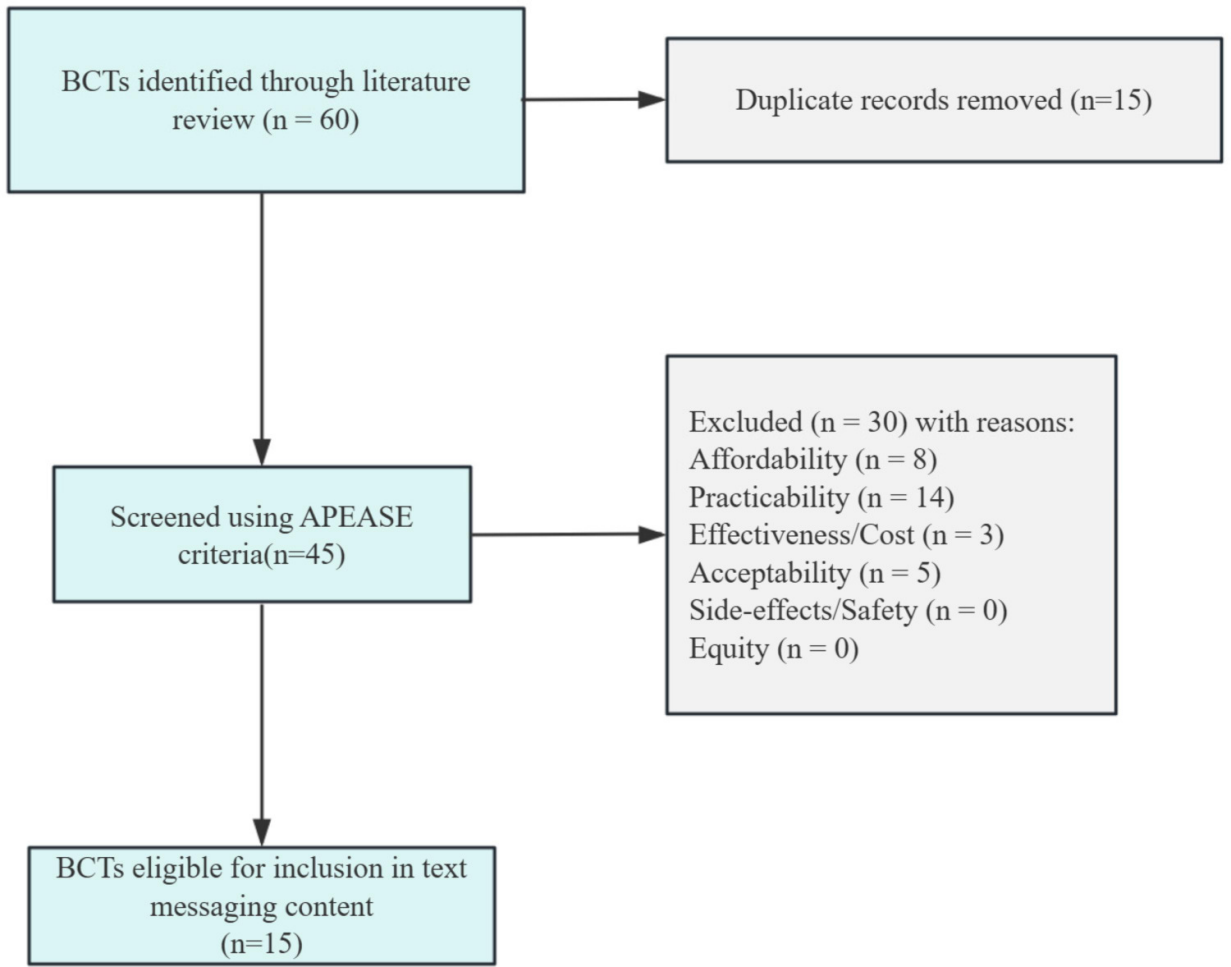
Diagram of BCTs identified in the literature and screened for inclusion.

Through a literature review, we initially included a total of 60 BCTs (see Supplementary File S2). After removing duplicates and screening for relevance, 45 potential BCTs were identified. These 45 BCTs were then evaluated using the APEASE criteria. Following this assessment, 30 BCTs were excluded. As a result, 15 BCTs were finally selected to address the identified barriers and facilitators in the text message content (Figure 3). These selected BCTs were subsequently incorporated into the BCW mapping tables (Supplementary File S1).

### Step 4. Message development

ZX and WL drafted the messages in Chinese based on the 15 BCTs identified in the previous stage. An initial bank of 115 messages was developed, with content distributed across disease and medication information, benefits of adherence, and harms of non-adherence (see Supplementary File S3). Each message was designed to correspond to a single BCT. All 115 messages were initially drafted by two medical experts and subsequently revised by a communication specialist. These revisions mainly involved minor changes in wording and punctuation to simplify the content and enhance the tone of engagement and support. The messages were further edited to ensure that the reading level did not exceed junior high school level.

After WWX completed the initial BCT coding, a consensus meeting was held between WWX and WL to finalize the BCT combinations assigned to each message. In total, 11 messages did not retain their originally intended BCTs (see Table 2), while 104 messages (90%) preserved their initial BCTs as planned.For the 11 messages where the original codes were not retained, at least one BCT was used for recoding to ensure conceptual consistency. Initially, “(1.7) Review outcome goal(s)” and “(4.1) Instruction on how to perform the behavior” were considered unsuitable for delivery via text messages, as ZX and WL believed that providing such instructions would be challenging to make acceptable or applicable to all participants. However, during the consensus process, some messages originally assigned to other BCTs were also coded as 4.1, and it was ultimately decided to include them. Overall, this iterative consensus process resulted in a finalized bank of 115 text messages designed to support medication adherence (see Supplementary File S3).

**Table 2:** Overview of behavior change technique alterations resulting from the fidelity check.

Message content	Initial coding	Final consensus coding
Even if your blood lipid levels are normal, you still need to take statins. These drugs help stabilize plaques and prevent unexpected events.	(5.1) Information about health consequences	(15.1) Verbal persuasion about capability
Taking antiplatelet medications may cause bleeding. Always watch for gum, nasal, gastrointestinal bleeding (black stools), or blood in urine. Seek medical attention promptly if any occur.	(15.1) Verbal persuasion about capability	(5.1) Information about health consequences
Clinically, most patients who experience recurrence are those who do not adhere to their medication regimen. Patients who take medication as prescribed tend to remain stable.	(6.2) Social comparison	(5.1) Information about health consequences
Statins may affect liver function, so liver function tests should be performed regularly.	(5.1) Information about health consequences	(15.1) Verbal persuasion about capability
Attend regular follow-up visits, as your doctor needs to adjust your treatment plan based on your condition.	(1.4) Action planning	(8.1) Behavioral practice/rehearsal
Most patients report that sticking to medication has improved their symptoms. Think about what changes adherence has brought you.	(15.1) Verbal persuasion about capability	(6.2) Social comparison
Ask yourself: Has sticking to medication relieved my chest tightness or pain?	(15.3) Focus on past success	(15.4) Self-talk
According to research, nearly one-third of post-PCI patients have poor medication adherence. Not taking medication within the first year increases cardiovascular event risk by 40% and death risk by 34%.	(5.6) Information about emotional consequences	(5.1) Information about health consequences
Stopping medication can worsen coronary conditions and increase the risk of angina and reinfarction.	(5.6) Information about emotional consequences	(5.1) Information about health consequences
Taking medication helps you recover gradually, giving you more time and energy to spend with your family and friends.	(5.6) Information about emotional consequences	(5.1) Information about health consequences
According to WHO, in the first year after a heart attack, 10 out of 100 people die, and 8 of these deaths could be prevented by long-term medication adherence.	(5.6) Information about emotional consequences	(5.1) Information about health consequences

## Discussion

In this study, we demonstrated a systematic application of the BCW to design a text message intervention aimed at improving medication adherence among patients following PCI. Given the substantial burden of recurrent cardiovascular events driven by poor medication adherence, there is an urgent need for scalable and cost-effective interventions to support sustained behavior change. Our intervention, MedText-PCI, directly addresses this gap by providing a structured, theory-driven approach to developing evidence-based text messages.

While prior studies have indicated that text messaging can be an effective and low-cost strategy to improve various health behaviors, including medication adherence, many interventions lack a clear description of their development process and often do not explicitly articulate the theoretical foundations underlying their content (11, 14). By contrast, our study offers a transparent and rigorous example of integrating behavior change theory throughout the design process.

The BCW framework enabled us to conduct a comprehensive behavioral diagnosis, identify relevant intervention functions, and systematically select behavior change techniques (BCTs) tailored to the barriers and facilitators specific to post-PCI patients. This approach ensured that each text message was purposefully crafted to address components of capability, opportunity, and motivation as outlined in the COM-B model (23). The use of APEASE criteria further strengthened the practical relevance of selected BCTs, taking into account feasibility, acceptability, and potential effectiveness.

A notable strength of our work lies in its multidisciplinary development process, involving medical experts and a communication specialist. This ensured clinical accuracy while maintaining simplicity and accessibility—critical factors for patient engagement and comprehension. By adapting all messages to a junior high school reading level, we addressed the well-documented influence of health literacy on medication adherence, which is often overlooked in digital health interventions (11).

Furthermore, our experience aligns with findings from studies applying the BCW in diverse contexts, such as gestational diabetes and lifestyle modification programs (26, 27). These studies similarly emphasize that a structured, theoretically grounded approach not only facilitates targeted intervention design but also strengthens the intervention's potential for real-world impact and scalability. However, using the BCW is resource- and time-intensive, and requires specialized expertise, which can pose challenges in routine clinical or community settings.

Several limitations should be acknowledged. First, the intervention was developed primarily through theoretical mapping and expert judgment, without direct patient involvement at this stage. While this approach is appropriate in early development, the lack of patient co-design may limit the cultural relevance and acceptability of the messages. To address this, patient perspectives will be incorporated in subsequent phases through qualitative research following the pilot study, and the findings will be used to refine the intervention. Second, while the COM-B model offers a robust framework, standardized measures to validate its predictive accuracy in real-world medication adherence remain lacking. Additionally, variations in individual factors such as age, digital literacy, and motivation may influence intervention effectiveness and should be explored in future studies. Therefore, conclusions should be limited to development outcomes such as the systematic BCW-guided process and the resulting message library rather than clinical impact. Effectiveness outcomes will be assessed in subsequent evaluation studies. Another limitation is the considerable time and complexity involved in applying the BCW. In our experience, the development process required extensive formative research, in-depth behavioral analysis, and multiple iterative design cycles, making it difficult for all members of the research team and stakeholders to remain consistently involved throughout. This limitation may reduce opportunities for broader input and co-creation, potentially affecting the intervention's relevance and acceptability from the perspective of patients and frontline healthcare providers.

Overall, this study underscores the importance of integrating behavioral theory and systematic frameworks such as BCW into digital health intervention design. By explicitly linking intervention components to theoretical mechanisms, we provide a foundation for robust evaluation and adaptation across different patient populations and health behaviors. For other chronic illnesses, development time may be reduced by using existing behavioural diagnosis data where available, retaining transferable BCT-informed message structures, and adapting only the disease-specific educational and motivational content through focused expert review and user testing.

In future trials, message frequency, timing, and personalization will be refined iteratively using pilot feasibility and acceptability data. Delivery schedules will be aligned with daily routines and medication times to enhance salience and reduce burden. Personalization will be guided by patient characteristics and reported barriers, such as regimen complexity, forgetfulness, concerns about adverse effects, and digital literacy, allowing the intervention to move from a standardized message library toward a more tailored delivery strategy while maintaining fidelity to the underlying behavior change mechanisms.

## Conclusion

This study demonstrated the feasibility and methodological rigor of using the BCW framework to develop a theory-based text messaging intervention aimed at improving medication adherence among post-PCI patients. Through this process, we developed a comprehensive library of 115 text messages incorporating 15 carefully selected BCTs, each targeting key barriers and facilitators specific to this high-risk population. The systematic BCT coding and fidelity checks enhance transparency and reproducibility, providing a robust foundation for future evaluations. The resulting message library holds promise not only for post-PCI patients but also for broader applications in other chronic disease contexts requiring long-term medication adherence. By clearly detailing the theoretical basis and active components of each message, this work enables future studies to investigate not only whether such interventions are effective but also why and how they work. Upcoming evaluations, including our planned pilot trial, will be crucial to confirm the intervention's impact on adherence and to guide optimization of content, timing, and delivery strategies. Ultimately, if proven effective, this intervention could offer a practical, scalable, and low-cost strategy to improve secondary prevention and reduce cardiovascular morbidity and mortality on a wider scale.

## Data Availability

The original contributions presented in the study are included in the article/Supplementary Material, further inquiries can be directed to the corresponding authors.
