# Supplementary material for: Using the behavior change wheel to develop text messages intervention (MedText-PCI) to promote medication adherence in patients after PCI
Source: Front Digit Health. 2026 May 8;8:1727102. doi: 10.3389/fdgth.2026.1727102 (PMC13194363; doi:10.3389/fdgth.2026.1727102)
Supplement: Supplementary file 1 [file Datasheet1.docx]

| **COM-B** | **Barriers and facilitators** | **Intervention function** | **Example BCTs** English version | **Example Message** |
| --- | --- | --- | --- | --- |
| Physical capability | Forgetfulness | Training | (1.2) Problem solving | Sometimes missing a dose is unavoidable, but don't be discouraged! More importantly, analyze the reasons, think about what went wrong, and prevent it from happening again. |
|  |  |  |  | If you miss a morning dose, make it up the same day; if not, do not take a double dose the next day. |
|  |  |  | (1.4) Action planning | If you often forget to take your medication, you can set an alarm or use a pillbox according to your preference. |
|  |  | Environmental restructuring | (7.1) Prompts/cues | Example: Mr./Ms. XX, please remember to take your medication on time. |
| Psychological capability | Lack of disease and medication knowledge | Education | (5.1) Information about health consequences | Why is regular follow-up necessary after stent implantation?   1. Stent surgery only treats severely narrowed vessels; mild or moderate lesions still carry risks of angina or heart attack.   (2) Even after a successful procedure, there is still a risk of recurrence; the opened vessel may become narrowed again. |
|  |  | Training | (1.2) Problem solving | If acute chest pain occurs again:   1. Stop activity and rest immediately.   (2) Take one nitroglycerin tablet under the tongue immediately. If ineffective, it may indicate an acute myocardial infarction—call emergency services and go to the hospital immediately. Even if relieved, seek medical evaluation as soon as possible. |
|  |  |  |  | If you are unsure about the purpose of your medications, refer to the PCI health manual, which has detailed information. |
|  |  | Persuasion | (15.1) Verbal persuasion about capability | Even if your blood lipid levels are normal, you still need to take statins. These drugs help stabilize plaques and prevent unexpected events. |
|  |  | Education | (5.1) Information about health consequences | Taking antiplatelet medications may cause bleeding. Always watch for gum, nasal, gastrointestinal bleeding (black stools), or blood in urine. Seek medical attention promptly if any of these occur. |
|  |  |  |  | Clinically, most patients who experience recurrence are those who do not adhere to their medication regimen. Patients who take medication as prescribed tend to remain stable, showing the importance of adherence. |
|  |  | Training | (4.1) Instruction on how to perform the behavior | Aspirin (antiplatelet agent)  Adverse reactions:  1 Gastrointestinal reactions, including nausea, vomiting, and upper abdominal discomfort.  2 Bleeding.  3 Allergic reactions, such as asthma, angioedema, or anaphylactic shock.  Precautions:  Take in the morning before meals to reduce gastric retention time and achieve maximum effectiveness. |
|  |  |  |  | Clopidogrel (Plavix)  Adverse reactions: Bleeding  Precautions:  1 Inform your doctor if you are using this medication before surgery.  2 Use with caution in patients with liver dysfunction.  3 Use with caution in patients with a bleeding tendency.  Additional notes: Take after breakfast in the morning. This medication may cause gastrointestinal mucosal damage. |
|  |  |  |  | Ticagrelor (Brilinta)  Adverse reactions: Bleeding  Precautions:  1 Inform your doctor if you are using this medication before surgery.  2 Use with caution in patients with liver dysfunction.  3 Use with caution in patients with a bleeding tendency. |
|  |  |  |  | Rivaroxaban (Xarelto)  Adverse reactions: Bleeding  Precautions:  1 Do not take with strong tea, beverages, or alcohol.  2 Use with caution in patients with a bleeding tendency. |
|  |  |  |  | Atorvastatin (Take after meals or before bedtime)  Effect: Lowers blood lipids and stabilizes plaques.  Precautions:  1 During treatment, cholesterol and creatine kinase levels should be checked regularly (monthly). Liver enzyme levels may increase during use; liver function tests should be monitored regularly.  2 Discontinue the medication if liver enzyme levels rise to three times the upper limit of normal, or if creatine kinase significantly increases, or if symptoms of myositis or pancreatitis occur. |
|  |  |  |  | Metoprolol (Betaloc) — a β-blocker  Effect: Lowers blood pressure, improves myocardial blood supply.  1 Reduces myocardial oxygen consumption by lowering myocardial contractility, heart rate, and blood pressure; prolongs diastole to improve coronary blood flow and perfusion, thereby reducing and relieving myocardial ischemia during daily activities or exercise, and improving quality of life.  2 Reduces infarct size, decreases fatal arrhythmias, and lowers acute phase mortality and cardiovascular event rates, including sudden cardiac death.  3 Long-term use improves long-term prognosis and increases survival rates. |
|  |  |  |  | Captopril  Effect: Lowers blood pressure.  Adverse reactions:  1 Rash, possibly accompanied by itching and fever, usually occurring within 4 weeks of treatment, appearing as maculopapular or urticarial rash; resolves with dose reduction, discontinuation, or antihistamines. 7%–10% may have eosinophilia or positive antinuclear antibodies.  2 Palpitations, tachycardia, chest pain.  3 Cough.  4 Altered taste. |
|  |  |  |  | Amlodipine (Norvasc)  Effect: Dilates peripheral arteries, relieves angina.  Adverse reactions:  1 Edema and headache.  2 Flushing and palpitations.  3 Hypotension.  Precautions: Monitor heart rate and blood pressure. |
|  |  |  |  | Isosorbide mononitrate (Imdur)  Effect: Dilates peripheral blood vessels.  Adverse reactions:  1 Headache, facial flushing.  2 Hypotension.  Precautions:  3 Can be taken whole or halved; do not chew or crush. |
|  |  |  |  | Bisoprolol (Concor) — a β-blocker  Effect: Lowers blood pressure and improves myocardial blood supply; used in the treatment of angina and myocardial infarction.  Adverse reactions: May include mild fatigue, chest tightness, dizziness, bradycardia, drowsiness, palpitations, headache, lower limb edema, diarrhea, constipation, nausea, abdominal pain, rash, itching, significant drop in blood pressure, slow pulse or atrioventricular block, tingling sensation or cold extremities, muscle weakness, painful muscle cramps, and reduced tearing. |
|  | Self-monitoring | Training | (2.3) Self-monitoring of behavior | Use a medication diary to evaluate your adherence. If there are missed doses, it means you need to work harder; if not, it shows you are doing great—please keep it up as it greatly benefits your health! |
|  | Integrating into daily habits | Training | (15.4) Self-talk | Link taking your medication with daily routines, such as asking yourself during morning brushing: Have I taken my medication today? |
|  |  |  | (7.1) Prompts/cues | Place your medication in a visible spot, such as on your bedside table. |
|  |  | Persuasion | (8.3) Habit formation | Take your medication at a fixed time every day. This helps you form a habit and makes it easier to remember. |
|  |  |  |  | Prepare your medications for the day when you get up, separating morning, noon, and evening doses and labeling them clearly to avoid forgetting. |
|  |  |  |  | Before and after each meal, ask yourself: Have I taken my medication? |
|  |  |  |  | By sticking to your medication routine, it will gradually become a habit. |
|  | Planning to overcome barriers | Environmental restructuring | (8.3) Habit formation | When going out, carry an emergency medicine kit (e.g., nitroglycerin). Also, do not forget to take aspirin and clopidogrel (or ticagrelor) after intervention surgery. |
|  |  |  |  | When traveling, prepare all your medications in advance and organize them into morning, noon, and evening sections in a pillbox. |
| Physical opportunity | Interference with daily life | Training | (1.4) Action planning | Life can be busy, and some people say work or travel causes them to forget medication. In this case, plan your medication schedule ahead of time and take it as planned. |
| Social opportunity | Regular follow-up | Training | (15.1) Verbal persuasion about capability | Statins may affect liver function, so liver function tests should be performed regularly. |
|  |  |  | (8.1)Behavioral practice/rehearsal | Attend regular follow-up visits, as your doctor needs to adjust your treatment plan based on your condition. |
|  | Social support | Training | (3.2) Social support (practical) | Sometimes you may forget to take your medication; think about who can remind and support you—perhaps your friends or family. |
|  |  |  |  | Many people find they are better able to adhere to medication under the supervision of family or friends. |
|  |  |  |  | There is an elderly patient, Mr. Wang, who is 75 years old. Five years after his stent surgery, he still adheres to his medication and follow-ups. If he can do it, so can you. |
|  |  |  |  | Your doctor wants you to share your feelings and any concerns so they can better help you. |
|  |  |  |  | If you have any questions about medications or other issues, contact us in time—we are happy to help you. |
|  |  |  | (15.1) Verbal persuasion about capability | Write down your medication questions and doubts, and give them to your doctor at your next visit. |
| Reflective motivation | Concerns about the future | Persuasion | (15.4) Self-talk | Remind yourself often that taking your medication helps you stay healthy and prevents disease progression. |
|  |  | Training | (15.1) Verbal persuasion about capability | Many patients say they worry about recurrence; the best way to prevent it is to adhere to your medication. |
|  |  | Enablement | (5.1) Information about health consequences | Medication can bring benefits, such as reducing hospitalizations and easing concerns about recurrence. |
|  | Perceived benefits and necessity | Persuasion | (15.1) Verbal persuasion about capability | Why continue medication after stent surgery? The stent only temporarily supports the treated vessel, and it does not treat other vessels. Medication helps prevent thrombosis in the stent and progression of atherosclerosis in other vessels. |
|  |  |  |  | Always remember: even if you feel better, do not stop your medication on your own! |
|  |  |  | (6.2) Social comparison | Most patients report that sticking to medication has improved their symptoms. Think about what changes adherence has brought you. |
|  |  |  | (15.4) Self-talk | Ask yourself: Has sticking to medication relieved my chest tightness or pain? |
|  |  |  | (5.1) Information about health consequences | According to research, nearly one-third of post-PCI patients have poor medication adherence. Not taking medication within the first year after PCI increases cardiovascular event risk by 40% and death risk by 34%. |
|  |  |  |  | Stopping medication can worsen coronary conditions and increase the risk of angina and reinfarction. |
|  |  |  |  | Taking medication helps you recover gradually, giving you more time and energy to spend with your family and friends. |
|  |  |  |  | According to WHO, in the first year after a heart attack, 10 out of 100 people die, and 8 of these deaths could be prevented by long-term medication adherence. |
|  |  |  | (9.2) Pros and cons | There may be many barriers to adherence, such as forgetfulness or side effects, but taking medication helps prevent recurrence. For your health, please stick to it! |
|  |  |  |  | What positive changes has medication adherence brought you? Fewer or no angina attacks? Improved test results? Feeling more energetic? |
|  | Self-efficacy | Persuasion | (1.1) Goal setting | Long-term adherence is challenging. Start with a small goal—stick to it for one week and give yourself a small reward when you succeed! |
|  |  | Education | (15.3) Focus on past success | You have stuck to it for 3 weeks. If you did not miss any doses, great job! If you did forget a few times, analyze the reasons and believe you can do better! |
|  |  |  |  | You have already persisted for 6 weeks. You can do it! Please keep it up—it's important for your condition. |
|  |  |  | (15.1) Verbal persuasion about capability | Understanding your treatment plan and medications is very important. Check the SMS you received in week 1 to review your medications, their effects, and side effects. |
|  |  | Persuasion | (1.7) Review outcome goal | Long-term adherence takes time. You may not have reached your goal yet, but you are on the right path. Keep going! |

## Chinese version

| **COM-B** | **障碍和促进因素** | **干预功能** | **BCT (CN)** | **短信** |
| --- | --- | --- | --- | --- |
| 身体能力 | 健忘 | 培训 | 解决问题 | 有时漏服药物是难以避免的，不要气馁!更重要的是分析原因，想一想哪里做的不到位，防止下次发生； |
|  |  |  |  | 若药物在早上发生漏服，则在当日补服；若当日未补服，切记不可在第二天补服。 |
|  |  |  | 行动计划 | 如果你忘记服药的情况时有发生，可以根据你的喜好采用设置一个服药闹钟、服药盒提醒等方法。 |
|  |  | 环境重构 | 提示/线索 | 示例：XX[先生/女士，请记得按时服用] |
| 心理能力 | 疾病药物知识缺乏 | 教育 | 关于健康后果的信息 | 支架术后为什么需要定期复查？ （1）支架手术仅处理高度狭窄的血管，轻中度病变的血管没有处理，仍有发生心绞痛或心肌梗死的危险； （2）支架手术虽然成功，但术后仍有一定的复发率，已经疏通的血管有再次狭窄的可能。 |
|  |  | 培训 | 解决问题 | 若急性胸痛再次发生： （1）停止活动，就地休息 （2）立即舌下含服硝酸甘油1片。如无效有可能发生了急性心肌梗死，应马上拨打急救电话，尽快去医院。如自行服药缓解，也应尽快去医院检查。 |
|  |  |  |  | 如果你不清楚你服用的药物的作用，打开PCI健康手册，里面有很详细的记录 |
|  |  | 说服 | 关于能力的口头说服 | 即使血脂正常也需要服用他汀类药物。这类药物可以稳定斑块，防止出现意外 |
|  |  | 教育 | 关于健康后果的信息 | 服用抗血小板药物可能导致出血，应时刻注意有无牙龈、鼻腔、消化道出血（黑便）、血尿等情况；若出现上述情况及时就医 |
|  |  |  |  | 临床上大多数复发的患者为不坚持服药的患者，而按时服药的患者病情相对稳定，可见遵医嘱服药的重要性 |
|  |  | 培训 | 关于如何执行一个行为的说明 | 您服用的药物：阿司匹林（抗血小板药） 不良反应： 1胃肠道反应，恶心、呕吐，上腹部不适 2出血 3过敏反应，哮喘，血管神经系统水肿或休克 注意事项：服用时间应为早上餐前服用，可减少药物在胃内的停留时间，发挥最大功效。 |
|  |  |  |  | 您服用的药物：氯吡格雷（波立维）： 不良反应：出血 注意事项： 1手术前使用该药应告知医生 2肝功能损害者慎用 3有出血倾向者慎用 注意事项：早上餐后服用，该药物具有胃肠道粘膜损害的副作用 |
|  |  |  |  | 您服用的药物：替格瑞洛（倍林达）： 不良反应：出血 注意事项： 1手术前使用该药应告知医生 2肝功能损害者慎用 3有出血倾向者慎用 |
|  |  |  |  | 您服用的药物：利伐沙班片（拜瑞妥） 不良反应：出血 注意事项： 1勿用浓茶，饮料，酒送服 2有出血倾向者慎用 |
|  |  |  |  | 您服用的药物：阿托伐他汀（餐后或睡前服用） 作用：降低血脂、稳定斑块 注意事项 1用药期间应定期（每个月）检查血胆固醇和血肌酸磷酸激酶。应用本品时血氨基转移酶可能增高，应定期监测肝功能试验。 2在本品治疗过程中如发生血氨基转移酶增高达正常高限的3倍，或血肌酸磷酸激酶显著增高或有肌炎、胰腺炎表现时，应停用本品。 |
|  |  |  |  | 您服用的药物：倍他乐克（美托洛尔）属于β受体阻滞剂 作用：降低血压、改善心肌供血 一是通过降低心肌收缩力、心率和血压, 使心肌耗氧量减少;同时延长心脏舒张期而增加冠脉及其侧支的血供和灌注, 从而减少和缓解日常活动或运动状态的心肌缺血发作, 提高生活质量。二是可缩小梗死范围, 减少致命性心律失常, 降低包括心脏性猝死在内的急性期病死率和各种心血管事件发生率。三是长期应用可改善患者的远期预后, 提高生存率。 |
|  |  |  |  | 您服用的药物：卡托普利： 作用:降低血压 不良反应： 1皮疹，可能伴有瘙痒和发热，常发生于治疗4周内，呈斑丘疹或荨麻疹，减量、停药或给抗组胺药后消失，7%～10%伴嗜酸性细胞增多或抗核抗体阳性。 2心悸，心动过速，胸痛 3咳嗽 4味觉迟钝 |
|  |  |  |  | 您服用的药物：洛活喜（苯硫磺氢氯地平片）： 作用：扩张外周动脉、缓解心绞痛 不良反应: 1、水肿和头痛 2、潮红、心悸 3、低血压 注意事项：监测心率和血压 |
|  |  |  |  | 您服用的药物：欣康片（单硝酸异山梨脂片）： 作用：扩张外周血管 不良反应： 1头痛、面部潮红 2低血压 注意事项： 3可整片或半片服用，不用嚼服或碾碎 |
|  |  |  |  | 您服用的药物：比索洛尔（康可）：属于β受体阻滞剂 作用：降低血压、改善心肌供血，用于心绞痛、心肌梗死的治疗。 不良反应：可见轻度乏力、胸闷、头晕、心动过缓、嗜睡、心悸、头痛和下肢水肿、腹泻、便秘、恶心、腹痛、红斑、瘙痒、血压明显下降、脉搏缓慢或房室传导阻滞、麻刺感或四肢冰凉、肌肉无力、肌肉痛性痉挛及泪少。 |
|  | 自我监测 | 培训 | 行为的自我监测 | 使用服药记录本去评估你的服药行为，如果存在漏服说明还需要努力，如果没有，说明你坚持得很好，请继续保持，这对你的疾病非常有益！ |
|  | 融入生活习惯 | 培训 | 自我对话 | 将服药和每天要做的事联系起来，比如早上刷牙时，问问自己，药物已经服用了吗？ |
|  |  |  | 提示/线索 | 把药物放在一个显眼的地方，比如床头柜上。 |
|  |  | 说服 | 形成习惯 | 每天在固定的时间服药，有助于您养成习惯，到时间就会想起来 |
|  |  |  |  | 每天起床提前把今天要服用的药物准备好，早中晚的药物放好并做好标识，到了服药的时候就不会忘记 |
|  |  |  |  | 在每次吃饭前、吃饭后，想一想，你的药已经吃过了吗？ |
|  |  |  |  | 坚持服药，慢慢地它会变成你的习惯。 |
|  | 做好应对障碍的计划 | 环境重构 | 解决问题 | 外出时应自备急救的药盒如硝酸甘油；另外介入术后服用的阿司匹林和氯吡格雷（替格瑞洛），千万不能忘记。 |
|  |  |  |  | 在外出旅游的时候，可以提前把药物都准备好，并放在服药盒，分为早、中、晚。 |
| 身体机会 | 日常生活的干扰 | 培训 | 行动计划 | 有时生活中会有许多事情，有的人说工作繁忙、外出旅游会导致忘记服药，针对这个情况可以提前计划好服药时间，时间到了就服药。 |
| 社会机会 | 定期随访 | 培训 | 关于能力的口头说服 | 他汀类药物可能会损害肝功能，故需定时复查肝功能指标。 |
|  |  |  | 行为形成 | 坚持定期随访，因为医生需要根据你的实际情况，调整药物治疗方案 |
|  | 社会支持 | 培训 | 社会支持(实际的) | 有时，你可能会忘记服药，想想谁能够经常给你提醒，给你提供帮助，可以是你的朋友或者你的家人。 |
|  |  |  |  | 许多人发现，在家人或朋友的监督下，自己更能坚持服药 |
|  |  |  |  | 有个老病人老王，他今年75岁，支架术后5年了仍然坚持规律服药和随访，他可以你也一定行 |
|  |  |  |  | 你的医生希望你告诉他们你的感受和你可能有的任何担忧，这样他们可更好地帮助你 |
|  |  |  |  | 有关药物或其他什么问题及时联系我们，我们非常乐意为你解答 |
|  |  |  | 关于口头能力的说服 | 写下你关于药物的问题和疑惑，下一次复查时交给医生。 |
| 反射性动机 | 对未来的担忧 | 说服 | 自我对话 | 经常告诉自己，坚持服药是为了让自己更健康，预防疾病恶化 |
|  |  | 培训 | 关于能力的口头说服 | 很多患者都说担心今后疾病复发，解决这最好的办法就是坚持服药 |
|  |  | 启发 | 提供健康益处的信息 | 药物能给你带来益处，比如说减少住院次数、让你不那么担心复发等 |
|  | 感知益处与必要性 | 说服 | 关于能力的口头说服 | 支架术后为何要继续服用药物？支架只能治好或者暂时支撑病变部位的血管，对其他血管没有治疗作用，目的在于防止支架内形成血栓、没放支架的血管动脉硬化继续恶化 |
|  |  |  |  | 时刻牢记，即使你自己感觉好多了，也不能擅自停药！ |
|  |  |  | 社会比较 | 大多数患者都说坚持服药让他们改善了，身体症状，想一想坚持服药给你带来了什么改变？ |
|  |  |  | 自我对话 | 问问自己，坚持服药是不是让我胸口闷，胸口疼的毛病缓解了？ |
|  |  |  | 关于健康后果的信息 | 据研究数据统计，近1/3的PCI术后患者药物依从性不佳，而PCI术后一年内不坚持服用药物会导致心血管事件风险增加40%，死亡风险增加34% |
|  |  |  |  | 如果停止服药，冠状动脉情况会更糟糕，心绞痛、再梗死的发生风险更高 |
|  |  |  |  | 服药可以帮助你慢慢恢复，让你有更多的时间和精力陪伴你的家人、朋友。 |
|  |  |  |  | 世界卫生组织数据显示，在心脏病发作后的第一年，100个人中就有10人死亡，其中8人可以通过长期坚持药物治疗挽救 |
|  |  |  | 利弊比较 | 坚持服药可能存在许多障碍比如说记性不好、服药后出现副作用，但它可以预防复发，因此坚持为了身体健康坚持服药吧！ |
|  |  |  |  | 坚持服药给您带来了哪些好的变化，是不是心绞痛发作次数减少甚至消失、各项指标趋向正常、感觉更有活力？ |
|  | 自我效能 | 说服 | 设定目标 | 长期坚持服药困难重重，可以从坚持服药1周这个小目标开始，完成了可以给自己一个小奖励！ |
|  |  | 教育 | 关注过去的成功 | 你已经坚持了3周，若你未发生漏服药物，那么你做的很棒，若你其中有几次忘记了服药，分析原因，相信你可以做的更好！ |
|  |  |  |  | 你已经坚持6周了，你可以做到，请继续保持，这对你病情稳定很重要！ |
|  |  |  | 关于能力的口头说服 | 了解自己的治疗方案和药物信息十分关键，看一看第1周收到的短信，你服用的药物有哪些以及它们的作用、副作用。 |
|  |  | 说服 | 回顾结果目标 | 长期坚持服药需要时间。可能你还没有达到你的目标，但你正在努力的过程中，继续保持！ |
