# Supplementary material for: Using the behavior change wheel to develop text messages intervention (MedText-PCI) to promote medication adherence in patients after PCI
Source: Front Digit Health. 2026 May 8;8:1727102. doi: 10.3389/fdgth.2026.1727102 (PMC13194363; doi:10.3389/fdgth.2026.1727102)
Supplement: Supplementary file 2 [file Datasheet2.docx]

**English version**

| **No.** | **Message Content** |
| --- | --- |
| 1 | Why do you need regular follow-up after stent implantation? (1) The stent only treats severely narrowed arteries. Mild or moderate lesions remain untreated and can still cause angina or myocardial infarction. (2) Even if the procedure is successful, there is still a risk of restenosis in the treated arteries. |
| 2 | If acute chest pain occurs again: (1) Stop activity and rest immediately. (2) Take one nitroglycerin tablet sublingually. If ineffective, it may indicate an acute myocardial infarction. Call emergency services immediately and go to the hospital as soon as possible. Even if relieved, still go to the hospital for evaluation. |
| 3 | If you are unsure about the purpose of your medications, check your PCI health handbook for detailed information. |
| 4 | Even if your lipid levels are normal, you still need to take statins. These medications help stabilize plaques and prevent unexpected events. |
| 5 | Taking antiplatelet drugs may cause bleeding. Watch for gum bleeding, nosebleeds, black stools, or blood in the urine. Seek medical attention promptly if these occur. |
| 6 | Most patients who relapse do so because they fail to take their medications consistently. Patients who take medications as prescribed generally have more stable conditions. This highlights the importance of adherence. |
| 7 | Your medication: Aspirin (antiplatelet drug) Adverse effects:  1. Gastrointestinal reactions: nausea, vomiting, upper abdominal discomfort 2. Bleeding 3. Allergic reactions: asthma, angioedema, or shock Instructions: Take in the morning before meals to reduce gastric retention time and maximize efficacy. |
| 8 | Your medication: Clopidogrel (Plavix) Adverse effects: Bleeding Instructions: 1. Inform your doctor before surgery if using this drug. 2. Use cautiously in patients with liver dysfunction. 3. Use cautiously in patients with bleeding tendencies. Take after breakfast as it may irritate the gastrointestinal mucosa. |
| 9 | Your medication: Ticagrelor (Brilinta) Adverse effects: Bleeding Instructions: 1. Inform your doctor before surgery if using this drug. 2. Use cautiously in patients with liver dysfunction. 3. Use cautiously in patients with bleeding tendencies. |
| 10 | Your medication: Rivaroxaban (Xarelto) Adverse effects: Bleeding Instructions: 1. Do not take with strong tea, beverages, or alcohol. 2. Use cautiously in patients with bleeding tendencies. |
| 11 | Your medication: Atorvastatin (take after meals or at bedtime) Function: Lowers lipids, stabilizes plaques Instructions: 1. Regularly monitor cholesterol and creatine kinase levels (monthly). Liver enzymes may increase, so liver function tests are needed. 2. Discontinue if liver enzymes increase to 3 times the upper limit of normal, or if creatine kinase significantly increases, or in case of myositis or pancreatitis symptoms. |
| 12 | Your medication: Metoprolol (Betaloc), a β-blocker Function: Lowers blood pressure, improves myocardial blood supply It reduces heart rate and contractility, lowering myocardial oxygen demand. It prolongs diastole, improving coronary perfusion, thus reducing ischemic episodes and improving quality of life. It also reduces infarct size, decreases fatal arrhythmias, lowers mortality, and improves long-term prognosis. |
| 13 | Your medication: Captopril Function: Lowers blood pressure Adverse effects: 1. Rash (may itch or have fever, often within 4 weeks), usually resolves after dose reduction, discontinuation, or antihistamines. 2. Palpitations, tachycardia, chest pain 3. Cough 4. Taste disturbances. |
| 14 | Your medication: Amlodipine (Norvasc) Function: Dilates peripheral arteries, relieves angina Adverse effects: 1. Edema and headache 2. Flushing, palpitations 3. Hypotension Instructions: Monitor heart rate and blood pressure. |
| 15 | Your medication: Isosorbide mononitrate (Imdur) Function: Dilates peripheral vessels Adverse effects: 1. Headache, facial flushing 2. Hypotension Instructions: Swallow whole or half tablet; do not chew or crush. |
| 16 | Your medication: Bisoprolol (Concor), a β-blocker Function: Lowers blood pressure, improves myocardial blood supply; used for angina and myocardial infarction Adverse effects: Mild fatigue, chest tightness, dizziness, bradycardia, drowsiness, palpitations, headache, leg edema, diarrhea, constipation, nausea, abdominal pain, rash, pruritus, significant hypotension, slow pulse or AV block, tingling or cold limbs, muscle weakness or cramps, and dry eyes. |
| 17 | Statins may impair liver function; regular liver function tests are required. |
| 18 | Regular follow-ups are necessary so your doctor can adjust your medication plan based on your condition. |
| 19 | Remind yourself that taking medication consistently keeps you healthier and prevents disease progression. |
| 20 | Many patients worry about recurrence. The best way to prevent it is to adhere to your medication regimen. |
| 21 | Medications bring benefits, such as fewer hospitalizations and less worry about recurrence. |
| 22 | Why continue medications after stent placement? Stents only treat the affected vessel segment and do not address other vessels. Medications prevent thrombosis inside the stent and further atherosclerosis. |
| 23 | Always remember: never stop medications on your own, even if you feel better! |
| 24 | Most patients report symptom improvement after adherence. Reflect on the positive changes medication has brought you. |
| 25 | Ask yourself: Has adherence reduced or relieved your chest tightness and pain? |
| 26 | Data shows that nearly one-third of PCI patients have poor adherence. Stopping medication in the first year increases cardiovascular event risk by 40% and death risk by 34%. |
| 27 | Stopping medications worsens coronary artery disease, increasing risks of angina and reinfarction. |
| 28 | Medication helps you gradually recover, giving you more time and energy to spend with family and friends. |
| 29 | According to global epidemiological data, approximately 5%–10% of patients die within the first year after myocardial infarction. Evidence indicates that long-term adherence to secondary prevention medications—including antiplatelet agents, statins, β-blockers, and ACE inhibitors or ARBs—significantly reduces the risk of recurrent events and mortality, suggesting that most cardiovascular deaths are preventable. |
| 30 | Although adherence can be difficult due to poor memory or side effects, it is essential for preventing recurrence and maintaining health. |
| 31 | What positive changes have you noticed? Fewer angina attacks, normal lab results, more energy? |
| 32 | Use a medication logbook to evaluate adherence. Missed doses mean improvement is needed; if none are missed, continue — it greatly benefits your condition. |
| 33 | Long-term adherence is challenging. Start with a one-week goal and reward yourself when achieved. |
| 34 | Link medication to daily routines, such as asking yourself after brushing teeth if you've taken your medicine. |
| 35 | Place medications in visible spots, like on the nightstand. |
| 36 | Take medications at a fixed time each day to develop a habit. |
| 37 | Prepare your medications in advance each morning for each dose and label clearly to avoid forgetting. |
| 38 | Before or after meals, ask yourself if you've taken your medication. |
| 39 | Over time, taking medication will become a habit. |
| 40 | If you often forget, think about who can remind and support you — family or friends. |
| 41 | Many people find it easier to adhere when supervised by family or friends. |
| 42 | Mr. Wang, 75 years old, has taken medications consistently for five years after PCI. You can do it too! |
| 43 | Tell your doctor about your feelings and concerns so they can help you better. |
| 44 | Contact us for any medication or other questions. We are happy to help. |
| 45 | Write down medication-related questions and give them to your doctor at your next visit. |
| 46 | Carry an emergency kit when going out, including nitroglycerin. Never forget aspirin and clopidogrel (or ticagrelor) after PCI. |
| 47 | When traveling, prepare and sort your medications in advance (morning, noon, evening) in a pillbox. |
| 48 | Missing doses is sometimes unavoidable. Don't be discouraged! Analyze the reasons and avoid next time. |
| 49 | If you miss a morning dose, take it the same day. Do not double up the next day. |
| 50 | If you frequently forget, consider setting medication alarms or using a pillbox reminder. |
| 51 | Busy work or travel can lead to missed doses. Plan ahead and take medication on time. |
| 52 | You've persisted for 3 weeks! If no missed doses, great job! If a few misses, analyze and improve. |
| 53 | You've persisted for 6 weeks! Keep going — it's important for stability. |
| 54 | Understanding your treatment and medications is crucial. Review the week 1 messages to recall your medications and their effects. |
| 55 | Long-term adherence takes time. You may not have reached your goal yet, but keep going! |
| 56 | Example: Mr./Ms. XX, please remember to take your medication on time. |
| 57 | Though not easy, every dose is an investment in your health. |
| 58 | Reviewing the positive changes from adherence can motivate you to continue. |
| 59 | Imagine fewer hospital stays and more time with family if you adhere. |
| 60 | If adherence feels overwhelming, focus on just completing today. |
| 61 | Reward yourself after several days without missing doses. |
| 62 | If side effects occur, consult your doctor — never stop on your own. |
| 63 | Create a reminder plan with family or friends to support you. |
| 64 | For travel, pre-sort medications into small boxes for convenience. |
| 65 | Choose a favorite song as a medication alarm to make it fun. |
| 66 | Reflect each night if you took medications properly and rate yourself. |
| 67 | Record feelings and symptom changes to strengthen adherence. |
| 68 | Place a reminder card at home saying 'Have you taken your medicine today?'. |
| 69 | Medication is self-care and self-responsibility. |
| 70 | Share your adherence experience with your doctor for support and encouragement. |
| 71 | Draw a smiley on the calendar each week you complete; more smiles, more achievement. |
| 72 | Plan daily medication times and link to routines (e.g., after breakfast). |
| 73 | Seeing stabilized blood pressure or lipids boosts confidence. |
| 74 | If setbacks occur, don’t give up; reflect and restart. |
| 75 | Imagine your condition worsening if you stop, as self-warning. |
| 76 | Talk to fellow patients for mutual encouragement. |
| 77 | Taking medications for your family is an act of love and responsibility. |
| 78 | Adherence benefits both today and future health. |
| 79 | Treat medication as a 'daily task' to enjoy the satisfaction of completion. |
| 80 | Silently say 'This is for my health' when taking medicine. |
| 81 | Keep water and medications by your bed for morning convenience. |
| 82 | Tell your doctor about your adherence during visits to show your efforts. |
| 83 | Feeling more energetic is the best proof — keep going! |
| 84 | Imagine your healthier, more secure self after one year of adherence. |
| 85 | When feeling weak, recall the satisfaction of past adherence. |
| 86 | Adherence reassures your family and reduces their worry. |
| 87 | Discuss your medication plan with family; their support strengthens you. |
| 88 | For business trips, prepare a checklist and pack medications in advance. |
| 89 | Adherence is the best support for your doctor’s treatment plan. |
| 90 | It can also reduce hospitalization costs and overall medical expenses. |
| 91 | Review your weekly adherence and set new goals for the next week. |
| 92 | Remind yourself 'I can do it' to reinforce confidence. |
| 93 | The more you understand about your medications, the easier it is to adhere. |
| 94 | If you miss a dose, don’t blame yourself. Adjust and keep going. |
| 95 | Imagine your future self thanking your current self for today's efforts. |
| 96 | Use different colored labels to distinguish multiple medications and avoid confusion. |
| 97 | If you need to take medication at night, prepare it in advance by the bed. |
| 98 | When your family sees your efforts, they will cheer you on. |
| 99 | Take photos to document your adherence journey. |
| 100 | Record improvements in a 'gratitude diary' to reinforce positive feelings. |
| 101 | Create a reward system and treat yourself after achieving milestones. |
| 102 | Pack medications in a small bag for easy access anytime. |
| 103 | Review goals and effects with your doctor regularly to stay motivated. |
| 104 | When facing challenges, review your adherence journey to regain strength. |
| 105 | Adherence reduces unexpected events such as heart attacks and rehospitalizations. |
| 106 | After each dose, say silently, 'I'm cheering for my health.' |
| 107 | Adherence cultivates patience and willpower. |
| 108 | Make medication a natural habit, like eating or washing your face. |
| 109 | Long-term adherence allows for more enjoyment of life. |
| 110 | Share your experience to inspire and help others. |
| 111 | Adherence is the best gift for your family. |
| 112 | Taking medication can set an example for others. |
| 113 | Set daily reminders on your phone for convenience. |
| 114 | Imagine your future self being grateful for your persistence today. |
| 115 | Adherence brings you closer to 'health freedom' — it is truly worth it. |

**Chinese version**

| **序号** | **短信内容** |
| --- | --- |
| 1 | 支架术后为什么需要定期复查？ （1）支架手术仅处理高度狭窄的血管，轻中度病变的血管没有处理，仍有发生心绞痛或心肌梗死的危险； （2）支架手术虽然成功，但术后仍有一定的复发率，已经疏通的血管有再次狭窄的可能。 |
| 2 | 若急性胸痛再次发生： （1）停止活动，就地休息 （2）立即舌下含服硝酸甘油1片。如无效有可能发生了急性心肌梗死，应马上拨打急救电话，尽快去医院。如自行服药缓解，也应尽快去医院检查。 |
| 3 | 如果你不清楚你服用的药物的作用，打开PCI健康手册，里面有很详细的记录 |
| 4 | 即使血脂正常也需要服用他汀类药物。这类药物可以稳定斑块，防止出现意外 |
| 5 | 服用抗血小板药物可能导致出血，应时刻注意有无牙龈、鼻腔、消化道出血（黑便）、血尿等情况；若出现上述情况及时就医 |
| 6 | 临床上大多数复发的患者为不坚持服药的患者，而按时服药的患者病情相对稳定，可见遵医嘱服药的重要性 |
| 7 | 您服用的药物：阿司匹林肠溶片（抗血小板药） 不良反应： 1胃肠道反应，恶心、呕吐，上腹部不适 2出血 3过敏反应，哮喘，血管神经系统水肿或休克 注意事项：服用时间应为早上餐前服用，可减少药物在胃内的停留时间，发挥最大功效。 |
| 8 | 您服用的药物：氯吡格雷（波立维）： 不良反应：出血 注意事项： 1手术前使用该药应告知医生 2肝功能损害者慎用 3有出血倾向者慎用 注意事项：早上餐后服用，该药物具有胃肠道粘膜损害的副作用 |
| 9 | 您服用的药物：替格瑞洛（倍林达）： 不良反应：出血 注意事项： 1手术前使用该药应告知医生 2肝功能损害者慎用 3有出血倾向者慎用 |
| 10 | 您服用的药物：利伐沙班片（拜瑞妥） 不良反应：出血 注意事项： 1勿用浓茶，饮料，酒送服 2有出血倾向者慎用 |
| 11 | 您服用的药物：阿托伐他汀（餐后或睡前服用） 作用：降低血脂、稳定斑块 注意事项 1用药期间应定期（每个月）检查血胆固醇和血肌酸磷酸激酶。应用本品时血氨基转移酶可能增高，应定期监测肝功能试验。 2在本品治疗过程中如发生血氨基转移酶增高达正常高限的3倍，或血肌酸磷酸激酶显著增高或有肌炎、胰腺炎表现时，应停用本品。 |
| 12 | 您服用的药物：倍他乐克（美托洛尔）属于β受体阻滞剂 作用：降低血压、改善心肌供血 一是通过降低心肌收缩力、心率和血压, 使心肌耗氧量减少;同时延长心脏舒张期而增加冠脉及其侧支的血供和灌注, 从而减少和缓解日常活动或运动状态的心肌缺血发作, 提高生活质量。 二是可缩小梗死范围, 减少致命性心律失常, 降低包括心脏性猝死在内的急性期病死率和各种心血管事件发生率。 三是长期应用可改善患者的远期预后, 提高生存率。 |
| 13 | 您服用的药物：卡托普利： 作用:降低血压 不良反应： 1皮疹，可能伴有瘙痒和发热，常发生于治疗4周内，呈斑丘疹或荨麻疹，减量、停药或给抗组胺药后消失，7%～10%伴嗜酸性细胞增多或抗核抗体阳性。 2心悸，心动过速，胸痛 3咳嗽 4味觉迟钝 |
| 14 | 您服用的药物：洛活喜（苯磺氢氯地平片）： 作用：扩张外周动脉、缓解心绞痛 不良反应: 1、水肿和头痛 2、潮红、心悸 3、低血压 注意事项：监测心率和血压 |
| 15 | 您服用的药物：欣康片（单硝酸异山梨脂片）： 作用：扩张外周血管 不良反应： 1头痛、面部潮红 2低血压 注意事项： 3可整片或半片服用，不用嚼服或碾碎 |
| 16 | 您服用的药物：比索洛尔（康可）：属于β受体阻滞剂 作用：降低血压、改善心肌供血，用于心绞痛、心肌梗死的治疗。 不良反应：可见轻度乏力、胸闷、头晕、心动过缓、嗜睡、心悸、头痛和下肢水肿、腹泻、便秘、恶心、腹痛、红斑、瘙痒、血压明显下降、脉搏缓慢或房室传导阻滞、麻刺感或四肢冰凉、肌肉无力、肌肉痛性痉挛及泪少。 |
| 17 | 他汀类药物可能会损害肝功能，故需定时复查肝功能指标。 |
| 18 | 坚持定期随访，因为医生需要根据你的实际情况，调整药物治疗方案 |
| 19 | 经常告诉自己，坚持服药是为了让自己更健康，预防疾病恶化 |
| 20 | 很多患者都说担心今后疾病复发，解决这最好的办法就是坚持服药 |
| 21 | 药物能给你带来益处，比如说减少住院次数、让你不那么担心复发等 |
| 22 | 支架术后为何要继续服用药物？支架只能治好或者暂时支撑病变部位的血管，对其他血管没有治疗作用，目的在于防止支架内形成血栓、没放支架的血管动脉硬化继续恶化 |
| 23 | 时刻牢记，即使你自己感觉好多了，也不能擅自停药！ |
| 24 | 大多数患者都说坚持服药让他们改善了，身体症状，想一想坚持服药给你带来了什么改变？ |
| 25 | 问问自己，坚持服药是不是让我胸口闷，胸口疼的毛病缓解了？ |
| 26 | 据研究数据统计，近1/3的PCI术后患者药物依从性不佳，而PCI术后一年内不坚持服用药物会导致心血管事件风险增加40%，死亡风险增加34% |
| 27 | 如果停止服药，冠状动脉情况会更糟糕，心绞痛、再梗死的发生风险更高 |
| 28 | 服药可以帮助你慢慢恢复，让你有更多的时间和精力陪伴你的家人、朋友。 |
| 29 | 全球流行病学数据显示，心肌梗死后的第一年内，约有 5%–10% 的患者死亡。研究表明，长期坚持二级预防药物治疗（如抗血小板药、他汀类、β受体阻滞剂及 ACEI/ARB 等）可 显著降低再发事件和死亡风险，提示多数心血管死亡是可预防的。 |
| 30 | 坚持服药可能存在许多障碍比如说记性不好、服药后出现副作用，但它可以预防复发，因此坚持为了身体健康坚持服药吧！ |
| 31 | 坚持服药给您带来了哪些好的变化，是不是心绞痛发作次数减少甚至消失、各项指标趋向正常、感觉更有活力？ |
| 32 | 使用服药记录本去评估你的服药行为，如果存在漏服说明还需要努力，如果没有，说明你坚持得很好，请继续保持，这对你的疾病非常有益！ |
| 33 | 长期坚持服药困难重重，可以从坚持服药1周这个小目标开始，完成了可以给自己一个小奖励！ |
| 34 | 将服药和每天要做的事联系起来，比如早上刷牙时，问问自己，药物已经服用了吗？ |
| 35 | 把药物放在一个显眼的地方，比如床头柜上。 |
| 36 | 每天在固定的时间服药，有助于您养成习惯，到时间就会想起来 |
| 37 | 每天起床提前把今天要服用的药物准备好，早中晚的药物放好并做好标识，到了服药的时候就不会忘记 |
| 38 | 在每次吃饭前、吃饭后，想一想，你的药已经吃过了吗？ |
| 39 | 坚持服药，慢慢地它会变成你的习惯。 |
| 40 | 有时，你可能会忘记服药，想想谁能够经常给你提醒，给你提供帮助，可以是你的朋友或者你的家人。 |
| 41 | 许多人发现，在家人或朋友的监督下，自己更能坚持服药 |
| 42 | 有个老病人老王，他今年75岁，支架术后5年了仍然坚持规律服药和随访，他可以你也一定行 |
| 43 | 你的医生希望你告诉他们你的感受和你可能有的任何担忧，这样他们可更好地帮助你 |
| 44 | 有关药物或其他什么问题及时联系我们，我们非常乐意为你解答 |
| 45 | 写下你关于药物的问题和疑惑，下一次复查时交给医生。 |
| 46 | 外出时应自备急救的药盒如硝酸甘油；另外介入术后服用的阿司匹林和氯吡格雷（替格瑞洛），千万不能忘记。 |
| 47 | 在外出旅游的时候，可以提前把药物都准备好，并放在服药盒，分为早、中、晚。 |
| 48 | 有时漏服药物是难以避免的，不要气馁!更重要的是分析原因，想一想哪里做的不到位，防止下次发生； |
| 49 | 若药物在早上发生漏服，则在当日补服；若当日未补服，切记不可在第二天补服。 |
| 50 | 如果你忘记服药的情况时有发生，可以根据你的喜好采用设置一个服药闹钟、服药盒提醒等方法。 |
| 51 | 有时生活中会有许多事情，有的人说工作繁忙、外出旅游会导致忘记服药，针对这个情况可以提前计划好服药时间，时间到了就服药。 |
| 52 | 你已经坚持了3周，若你未发生漏服药物，那么你做的很棒，若你其中有几次忘记了服药，分析原因，相信你可以做的更好！ |
| 53 | 你已经坚持6周了，你可以做到，请继续保持，这对你病情稳定很重要！ |
| 54 | 了解自己的治疗方案和药物信息十分关键，看一看第1周收到的短信，你服用的药物有哪些以及它们的作用、副作用。 |
| 55 | 长期坚持服药需要时间。可能你还没有达到你的目标，但你正在努力的过程中，继续保持！ |
| 56 | 示例：XX[先生/女士，请记得按时服用] |
| 57 | 坚持服药虽然不易，但每次坚持都是对健康的投资。 |
| 58 | 回顾一下过去坚持服药带来的积极变化，会让你更有动力继续坚持。 |
| 59 | 想象未来如果坚持服药，你会更少住院、更多时间陪伴家人。 |
| 60 | 如果感觉服药负担大，可以尝试一次只关注完成一天的目标。 |
| 61 | 如果连续几天都没有忘记服药，可以给自己一些小奖励，比如一个小礼物。 |
| 62 | 如果有副作用困扰，及时与医生沟通，不要擅自停药。 |
| 63 | 和朋友或家人一起制定服药提醒计划，让他们也成为你的监督者。 |
| 64 | 如果旅途中携带药物不方便，可以提前分装到小药盒中，方便随身携带。 |
| 65 | 选择一首喜欢的歌作为服药的提示铃声，增加坚持的乐趣。 |
| 66 | 每天晚上回顾一下当天是否已正确服药，并为自己打分。 |
| 67 | 记录服药后感觉的变化，例如精神状态或症状改善，帮助增强坚持的信心。 |
| 68 | 在家里明显的位置贴一张「今天吃药了吗？」的提醒卡片。 |
| 69 | 服药是一种照顾自己的方式，是对自己负责的表现。 |
| 70 | 告诉医生你的坚持经历，他们会为你点赞，也可能会给出更多支持。 |
| 71 | 每完成一周服药，就在日历上画一个小笑脸，累计的笑脸越多，成就感越大。 |
| 72 | 计划好每天服药的时间，最好固定与日常活动（如早餐后）相结合。 |
| 73 | 发现服药后血压、血脂等指标稳定，会让你更有信心。 |
| 74 | 如果中途出现挫折，不要气馁，及时总结并重新开始。 |
| 75 | 想象如果一直不服药，疾病可能会加重，这是一种自我警示。 |
| 76 | 可以和同样经历的病友交流经验，互相鼓励共同坚持。 |
| 77 | 为家人和爱的人坚持服药，是对他们的责任和爱。 |
| 78 | 坚持服药不仅为了今天，更是为了未来健康的每一天。 |
| 79 | 如果觉得麻烦，可以把服药想象成「每日任务」，完成后享受「打卡」的成就感。 |
| 80 | 每次拿药时，默念「这是为了我的健康」。 |
| 81 | 在床头准备水和药物，方便早晨第一时间服用。 |
| 82 | 每次复诊时，告诉医生你坚持服药的情况，让医生知道你的努力。 |
| 83 | 如果服药让你感觉更有活力，那就是最好的证明，坚持下去吧！ |
| 84 | 想象完成一年服药计划后，自己会更有安全感、更健康。 |
| 85 | 当觉得无力时，回忆之前坚持服药成功时的满足感。 |
| 86 | 坚持服药能让家人更安心，减少他们的担心。 |
| 87 | 和家人讨论你的服药计划，他们的理解与参与会让你更有力量。 |
| 88 | 如果出差或旅行前觉得麻烦，提前写好清单，一次性准备好药物。 |
| 89 | 坚持服药其实也是对医生治疗方案的最大配合。 |
| 90 | 坚持用药也可以减少住院的经济负担，降低整体医疗费用。 |
| 91 | 每周末总结一周的服药情况，为下周制定新的目标。 |
| 92 | 每一次提醒自己「我做得到」，帮助强化信心。 |
| 93 | 了解更多药物知识，越了解越能坚持。 |
| 94 | 若偶尔漏服，不要责怪自己，重要的是立即调整，持续前进。 |
| 95 | 想象未来的自己感谢现在努力坚持的自己。 |
| 96 | 如果同时服用多种药物，可以给每种药贴上不同颜色的小标签，避免混淆。 |
| 97 | 如果夜里需要服药，可提前准备好水和药放在床头柜。 |
| 98 | 当家人看到你的努力，也会为你加油打气。 |
| 99 | 坚持服药时，可以拍照记录，形成「坚持相册」。 |
| 100 | 如果服药后症状好转，可记录「感谢日记」强化好感受。 |
| 101 | 设计自己的「服药奖励机制」，每坚持一段时间就犒劳自己一次。 |
| 102 | 可以把药物装在小包里，随时随地都能方便服用。 |
| 103 | 定期和医生回顾服药目标和效果，保持对进展的期待。 |
| 104 | 当遇到困难时，回顾之前坚持的历程，重新获得力量。 |
| 105 | 坚持服药帮助减少意外事件的发生，如心梗、再次住院等。 |
| 106 | 每次完成服药后，可以默默说一句「我为健康加油」。 |
| 107 | 坚持不仅改变了身体，更能培养耐心和意志力。 |
| 108 | 让服药成为一种生活习惯，就像吃饭、洗脸一样自然。 |
| 109 | 如果长期保持，未来会有更多享受美好生活的机会。 |
| 110 | 和朋友分享坚持经验，也许能帮助更多人。 |
| 111 | 坚持服药是对家人最好的礼物。 |
| 112 | 服药不仅是个人行动，也可以成为影响周围人的榜样。 |
| 113 | 可以在手机备忘录里设置每日提醒，更精准方便。 |
| 114 | 想象多年后，身体依然健康的自己，感谢今天坚持不懈的努力。 |
| 115 | 坚持服药让你离「健康自由」更近一步，这是最值得的坚持。 |
