# Supplementary material for: Using the behavior change wheel to develop text messages intervention (MedText-PCI) to promote medication adherence in patients after PCI
Source: Front Digit Health. 2026 May 8;8:1727102. doi: 10.3389/fdgth.2026.1727102 (PMC13194363; doi:10.3389/fdgth.2026.1727102)
Supplement: Supplementary file 3 [file Datasheet3.docx]

# BCT taxonomy for improving medication adherence (including intervention functions)

| **BCT（Chinese）** | **BCT(English)** | **Intervention Functions** |
| --- | --- | --- |
| 关于健康后果的信息 | (5.1) Information about health consequences | Education, Persuasion |
| 提供健康益处的信息 | (5.6) Information about health benefits | Education, Persuasion |
| 指导如何执行行为 | (4.1) Instruction on how to perform the behaviour | Education, Training |
| 信息关于先行事件 | (4.2) Information about antecedents | Education |
| 行动计划 | (1.4) Action planning | Training, Enablement |
| 设定行为目标 | (1.1) Goal setting (behaviour) | Training, Enablement |
| 回顾行为目标 | (1.5) Review behaviour goal(s) | Training, Enablement |
| 问题解决 | (1.2) Problem solving | Training, Enablement |
| 行为契约 | (1.8) Behavioral contract | Enablement, Persuasion |
| 承诺 | (1.9) Commitment | Enablement, Persuasion |
| 比较利弊 | (9.2) Pros and cons | Persuasion, Education |
| 提示/线索 | (7.1) Prompts/cues | Environmental restructuring, Enablement |
| 减少提示 | (7.3) Reduce prompts/cues | Environmental restructuring |
| 重塑物理环境 | (12.1) Restructuring the physical environment | Environmental restructuring |
| 重塑社会环境 | (12.2) Restructuring the social environment | Environmental restructuring |
| 添加环境对象 | (12.5) Adding objects to the environment | Environmental restructuring |
| 行为形成 | (8.1) Behavioural practice/rehearsal | Training |
| 行为替代 | (8.2) Behaviour substitution | Training, Enablement |
| 形成习惯 | (8.3) Habit formation | Training, Enablement |
| 生动展示行为 | (6.1) Demonstration of the behaviour | Modelling |
| 社会比较 | (6.2) Social comparison | Persuasion, Modelling |
| 他人批准信息 | (6.3) Information about others’ approval | Persuasion |
| 自我监测行为 | (2.3) Self-monitoring of behaviour | Enablement |
| 监测行为结果 | (2.7) Feedback on outcome(s) of behaviour | Enablement |
| 反馈行为 | (2.2) Feedback on behaviour | Enablement |
| 生物反馈 | (2.6) Biofeedback | Enablement |
| 生理/健康监测 | (2.5) Monitoring outcome(s) by others | Enablement |
| 社会支持（实际的） | (3.2) Social support (practical) | Enablement |
| 社会支持（非特指） | (3.1) Social support (unspecified) | Enablement |
| 口头能力说服 | (15.1) Verbal persuasion about capability | Persuasion |
| 心理排练 | (15.2) Mental rehearsal of successful performance | Training |
| 聚焦过去成功 | (15.3) Focus on past success | Persuasion |
| 自我对话 | (15.4) Self-talk | Persuasion |
| 身份识别塑造 | (13.1) Identification of self as role model | Modelling |
| 重构信念 | (13.2) Framing/reframing | Persuasion |
| 不兼容信念 | (13.3) Incompatible beliefs | Persuasion |
| 重视身份认同 | (13.4) Valued self-identity | Persuasion |
| 行为相关身份 | (13.5) Identity associated with changed behaviour | Persuasion |
| 社会奖励 | (10.4) Social reward | Incentivisation |
| 自我奖励 | (10.9) Self-reward | Incentivisation |
| 物质奖励 | (10.1) Material incentive (behaviour) | Incentivisation |
| 社会激励 | (10.5) Social incentive | Incentivisation |
| 非特指激励 | (10.6) Non-specific incentive | Incentivisation |
| 自我激励 | (10.7) Self-incentive | Incentivisation |
| 行为结果激励 | (10.8) Incentive (outcome) | Incentivisation |
| 情景特定奖励 | (10.6) Situation-specific reward | Incentivisation |
| 删除奖励 | (14.9) Reduce reward frequency | Coercion |
| 情境代价 | (14.1) Behaviour cost | Coercion |
| 惩罚 | (14.2) Punishment | Coercion |
| 奖励近似 | (14.4) Reward approximation | Incentivisation |
| 奖励完成 | (14.5) Rewarding completion | Incentivisation |
| 惩罚移除 | (14.10) Remove punishment | Coercion |
| 身体变化 | (12.6) Body changes | Environmental restructuring |
| 想象惩罚 | (16.1) Imaginary punishment | Persuasion |
| 想象奖励 | (16.2) Imaginary reward | Persuasion |
| 替代行为奖励 | (14.8) Reward alternative behaviour | Incentivisation |
| 非兼容行为奖励 | (14.7) Reward incompatible behavior | Incentivisation |
| 情绪后果信息 | (5.6) Information about emotional consequences | Persuasion |
| 预期后悔 | (5.5) Anticipated regret | Persuasion |
| 降低负面情绪 | (11.2) Reduce negative emotions | Enablement |
